# Giant renal oncocytoma: a case report and review of the literature

**DOI:** 10.1186/1752-1947-4-52

**Published:** 2010-02-17

**Authors:** Sami Akbulut, Ayhan Senol, Bahri Cakabay, Arsenal Sezgin

**Affiliations:** 1 Department of Surgery, Diyarbakir Education and Research Hospital, Op Dr Seref Inaloz Caddesi, 21400, Diyarbakir, Turkey; 2 Department of Radiology, Diyarbakir Education and Research Hospital, Op Dr Seref Inaloz Caddesi, 21400, Diyarbakir, Turkey; 3 Department of Pathology, Diyarbakir Education and Research Hospital, Op Dr Seref Inaloz Caddesi, 21400, Diyarbakir, Turkey

## Abstract

**Introduction:**

Renal oncocytomas are benign neoplasms derived from cells of the distal renal tubule, and comprise 5% to 7% of primary renal neoplasms. Oncocytomas are mostly asymptomatic, and the majority of tumors are discovered incidentally. In this case report, we present a case of a patient with a giant oncocytoma arising from her left kidney.

**Case presentation:**

We describe a 25-year-old Turkish woman who was admitted to our hospital with abdominal pain and a 3-year palpable abdominal mass, which was found present since her second pregnancy. Examination revealed a 15 × 20-cm mass in her abdominal cavity. Computed tomography revealed a mass with regular outlines, measuring 18 × 11 × 12 cm, associated with the left kidney, and causing marked hydroureteronephrosis. We excised the mass and performed a left nephrectomy on our patient. The immunohistopathology of the mass was consistent with renal oncocytoma. No local or distant metastasis was seen at 6 months postoperatively.

**Conclusion:**

To the best of our knowledge, this is the second largest renal oncocytoma described in the English language literature. This is also the first reported giant oncocytoma that presented during pregnancy.

## Introduction

Oncocytomas are benign, nonurothelial, epithelial tumors that constitute 5% to 7% of primary renal neoplasms. Classically, an oncocytoma is a solid mass that develops in the renal parenchyma and has a central fibrous scar [1,2]. Most are asymptomatic at presentation and are discovered only incidentally during evaluation of non-urological problems, but hematuria and pain occur in a minority of documented cases [3].

## Case presentation

**Figure 1:**
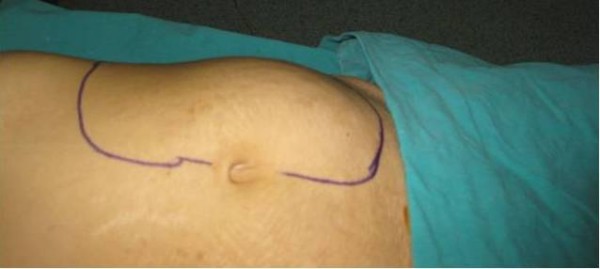
**Preoperative view of the mass**.

A 25-year-old Turkish woman was admitted to our hospital in October 2008. She was suffering from abdominal pain, weakness, anorexia, and a 7-kg to 8-kg weight loss. It was discovered that she had a palpable abdominal mass three years prior to presentation. Physical examination revealed a 15 × 20-cm mass with regular contours in the her periumbilical region (Figure 1). Four years prior to presentation, during her second pregnancy, she was admitted to a hospital because of abdominal pain, and an ultrasonographic examination then revealed an abdominal mass. As she did not want to terminate her pregnancy, she declined to undergo further examinations. After her pregnancy, she was told by her doctor that the mass had grown markedly. Because she was afraid of regularly visiting a doctor, she treated herself using alternative medicines such as eucalyptus tea, bay leaves and oleander. She claimed that drinking this tea made the mass shrink.

**Figure 2:**
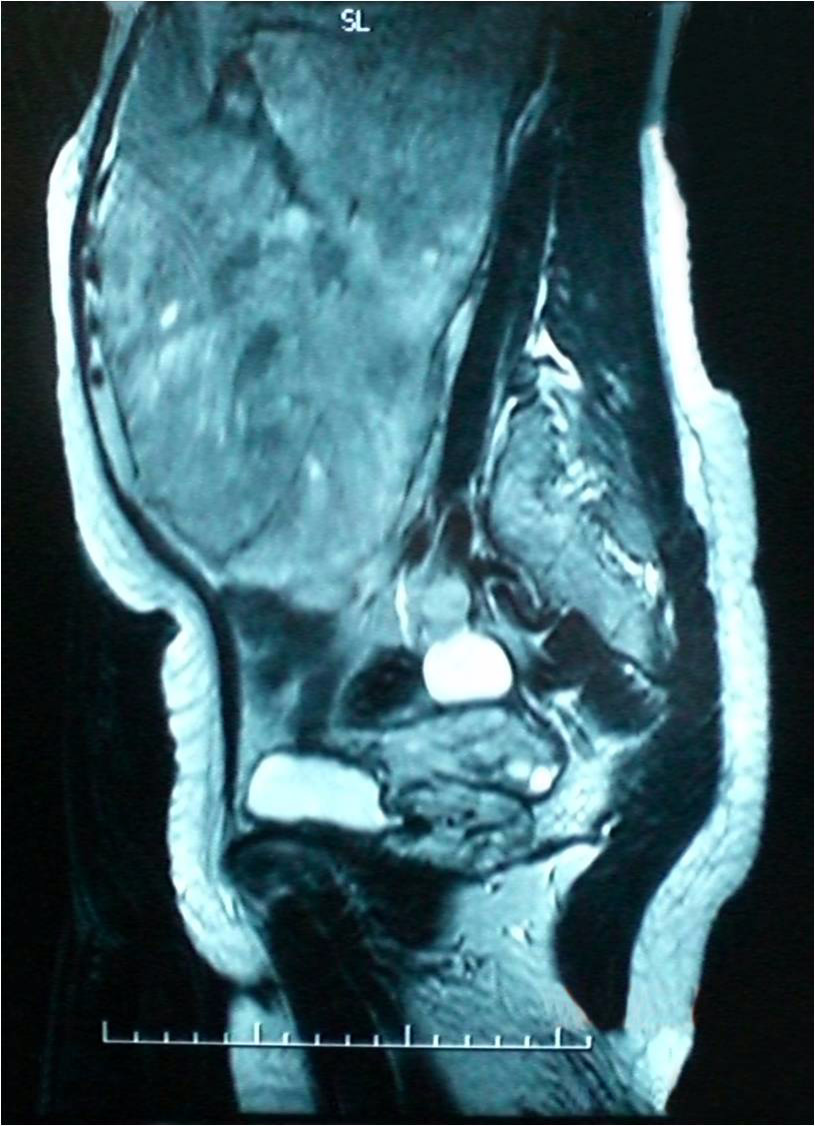
**T1-weighted coronal magnetic resonance imaging shows a huge heterogenous intra-abdominal mass and anterolateral displacement of the left ureter**.

**Figure 3:**
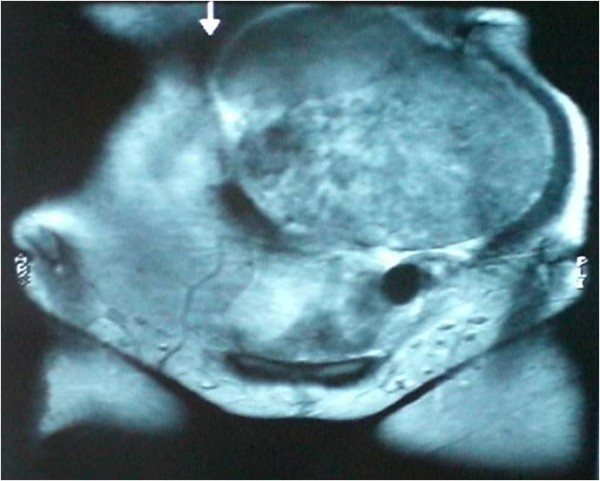
**T2-weighted sagittal magnetic resonance imaging shows a huge heterogenous intra-abdominal mass and anterolateral displacement of the left ureter**.

**Figure 4:**
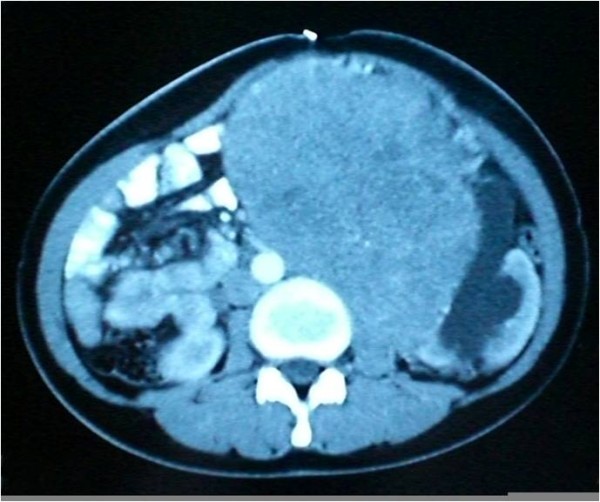
**A contrast-enhanced axial computed tomography image shows a huge, capsulated, heterogeneously enhancing mass originating from the lower pole of the left kidney**. Note the moderate hydronephrosis and lateral displacement of the left ureter.

**Figure 5:**
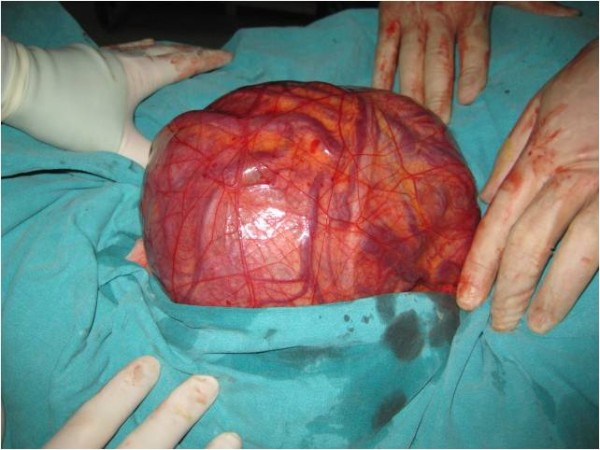
**The appearance of the mass after the midline incision, which shifted the ureter anterolaterally**.

**Figure 6:**
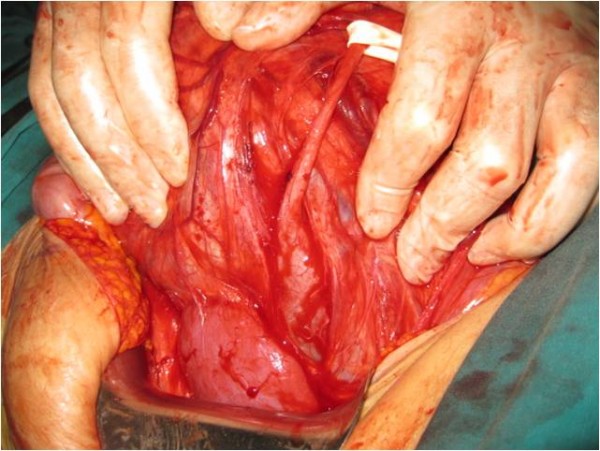
**The relationship between the mass and the kidney**.

**Figure 7:**
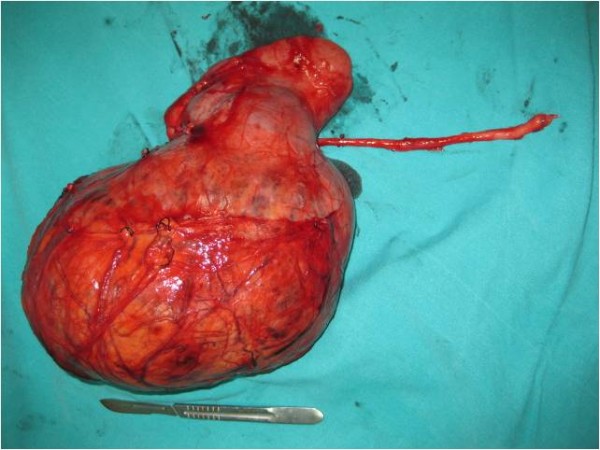
**The appearance of the mass with the kidney after resection**.

Her laboratory results at the time of presentation were as follows: hemoglobin, 13 g/dl; white blood cell count, 10,500/cm^3^; platelets, 302,000/cm^3^; sodium, 135 mEq/L; potassium, 4.1 mEq/L; blood urea nitrogen, 28 mg/dl; creatinine, 1.1 mg/dl; glucose, 110 mg/dl; aspartate aminotransferase (AST), 36 IU/L; and alanine aminotransferase (ALT), 41 IU/L. On abdominal X-rays, all of her intestines were seen shifted to the right. An intravenous pyleogram (IVP) showed a mass associated with her left kidney. Abdominal computed tomography (CT) and magnetic resonance imaging (MRI) revealed an 18 × 11 × 12-cm mass located in the middle of her left kidney and extending inferiorly, with diffuse contrast enhancement (Figures 2 to 3). The mass was consistent with renal cell carcinoma on CT. Owing to the mass, she develped left hydroureteronephrosis (Figure 4). Her right kidney appeared to be within normal limits on the IVP and CT examinations. A laparotomy was performed via a midline incision. A 25 × 16 × 12 cm, 3380 g mass, arising from the left kidney and shifting the ureter proximally, was excised together with the left kidney (Figures 5, 6, 7). Our patient was discharged on the sixth postoperative day, with no signs of complications.

Meanwhile, microscopic examination of hematoxylin and eosin (H&E)-stained sections at low power showed a neoplasm with a pseudocapsule and a tubulocystic pattern divided by thin fibrous septa. At higher magnification, the cells appeared to be round to polygonal with abundant granular eosinophilic cytoplasm, a regular round nucleus, and a central nucleolus. There were no foci of clear cell changes, necrosis, or mitotic figures.

## Discussion

Renal oncocytoma was initially described in 1942 by Zippel. Oncocytomas are benign tumors of the kidney that are usually diagnosed postoperatively, after an erroneous diagnosis of renal cell carcinoma (RCC).

A summary of the studies about giant oncocytomas published in the English literature is presented as Additional File 1. To date, Demos *et al*. [4] have reported the largest and heaviest oncocytoma, which measured 27 × 20 × 15 cm and weighed 4652 g and Sundararajan *et al*. [5] reported the second heaviest renal oncocytoma (3353 g, 20 cm in size). Meanwhile, Banks *et al*. [6] reported the third heaviest renal oncocytoma (3090 g, 21 × 18 × 15 cm) and Kilic *et al*. [7] reported the fourth heaviest oncocytoma (2680 g, 20 × 15 × 10 cm). We report what we believe is now the second largest renal oncocytoma, a mass weighing 3380 g and measuring 25 × 15 × 12 cm.

Renal oncocytomas are uncommon and consist of a pure population of oncocytes, which are large well-differentiated neoplastic cells with intensely eosinophilic granular cytoplasm due to the large number of mitochondria [8-10]. Like chromophobe carcinomas, oncocytomas appear to originate from collecting duct cells [9]. In most cases, oncocytomas and different histological subtypes of RCC can be differentiated on gross inspection and from H&E-stained microscopic slides. Sometimes the differentiation is difficult, especially that among the eosinophilic variant of chromophobe RCC, the granular variant of conventional RCC, and oncocytoma [11,12].

The most useful marker for the differentiation of renal tumors are vimentin (positive in conventional renal cell carcinoma and negative in chromophobe cell carcinoma and oncocytoma), CK7 (positive in chromophobe cell carcinoma and negative in oncocytoma and conventional renal cell carcinoma), RCC marker and CD10 (positive in conventional renal cell carcinoma and negative in chromophobe cell carcinoma and oncocytoma), and Hale's colloidal iron staining with diffuse reticular pattern and perinuclear halo (which is present in chromophobe cell carcinoma but absent in oncocytoma and conventional renal cell carcinoma) [13-16]. The distal nephron proteins claudin-7 and claudin-8 have potential use as immunohistochemical biomarkers in the differential diagnosis of chromophobe renal cell carcinoma and oncocytoma [13]. Homogeneous epithelial cell adhesion molecule (EpCAM) expression confirms the diagnosis of chromophobe carcinoma rather than oncocytoma.

Oncocytomas can appear on CT and ultrasound as a central scar located within a homogeneous, well-circumscribed solid mass [17]. However, this is considered nonspecific and occurs in only 33% of cases of oncocytomas [2,3]. In addition, this finding does not exclude clear cell carcinoma. In our case, no such central scar was seen on CT. Furthermore, the mass was hypervascular. As such, differentiating oncocytoma and RCC in preoperative imaging studies remains difficult. Moreover, RCC can coexist with oncocytoma.

Renal oncocytomas are almost invariably benign and no cases of metastasis have been reported. Even when very large, they are generally well encapsulated and are rarely invasive or associated with metastases [18]. In our case, no relapse occurred in the first 6 months postoperatively. This benign nature has important therapeutic implications, and oncocytoma should be considered in the differential diagnosis of patients with small renal masses discovered incidentally or with tumors found within a solitary kidney. Despite their benign behavior, however, oncocytomas should be monitored closely and treated if there is evidence of rapid growth or a coexisting RCC, which occurs in 10% to 32% of reported patients [19], because progressive growth can destroy the adjacent renal parenchyma and may constitute another indication for therapeutic intervention [20].

Renal oncocytoma has a benign clinical course with excellent long-term outcomes. Surgical treatment of renal tumors is still unclear. Although radical surgery has been employed in the past as principal therapy, more precise preoperative and peri-operative diagnosis should allow more frequent use of conservative surgery, such as partial nephrectomy or tumor excision. Conservative management such as organ-sparing surgery or partial nephrectomy should be reserved for bilateral tumors or where the tumor occurs in a solitary kidney [16,19].

## Conclusion

Although there have been many improvements in the histopathological diagnosis of giant oncocytoma, more case reports and studies are needed to understand the behavior, prognosis and treatment of this tumor.

## Abbreviations

RCC: renal cell carcinoma; EpCAM: epithelial cell adhesion molecule; CT: computed tomography; IVP: intravenous pyleogram; MRI: magnetic resonance imaging.

## Consent

Written informed consent was obtained from our patient for publication of this case report and any accompanying image. A copy of the written consent is available for review by the Editor-in-Chief of this journal.

## Competing interests

The authors declare that they have no competing interests.

## Authors' contributions

SA and BC performed the surgical procedure, contributed in writing the article and review of the literature, and undertook a comprehensive literature search. SA contributed in the design of the study and preparation of the manuscript. ARS provided the histopathological information and AS provided the radiological information. All authors read and approved the final manuscript

## Supplementary Material

Additional file 1 **Table S1**. The summary of the studies about giant oncocytoma published in the English language literature. Click here for file
