# Supplementary material for: Giant renal oncocytoma: a case report and review of the literature
Source: J Med Case Rep. 2010 Feb 17;4:52. doi: 10.1186/1752-1947-4-52 (PMC2827435; doi:10.1186/1752-1947-4-52)
Supplement: Additional file 1 — Table S1. The summary of the studies about giant oncocytoma published in the English language literature. [file 1752-1947-4-52-S1.DOC]

| **References** | **Date** | **Age** | **Sex** | **Location** | **Complain** | **Surgical procedure** | **Size(cm)** | **Weight (gr)** | **Pathology** | **Complication** |
| --- | --- | --- | --- | --- | --- | --- | --- | --- | --- | --- |
| Kilic^7^ | 2003 | 65 | M | Left kidney | Abdominal pain | Radical Nephrectomy | 20*15*10 | 2680 | Oncocytoma | None found |
| Ponholzer^21^ | 2002 | 63 | F | Bilateral kidney | Weight loss |  | L:11*8.5*12 R:16*12*15 | | Oncocytoma | None found |
| Sundararajan^5^ | 2008 | 37 | M | Right kidney | Abdominal mass and moderate hypertension | Radical Nephrectomy | 20 | 3353 | Oncocytoma | Hypertension resolved |
| Demos^4^ | 1988 | 64 | M | Right kidney | Abdominal mass | Radical Nephrectomy | 27*20*15 | 4652 | Oncocytoma | None found |
| Banks^6^ | 2001 | 57 | M | Right kidney | Abdominal pain | Radical Nephrectomy | 21*18*15 | 3090 | Oncocytoma | None found |
| Haddad^22^ | 1992 | 23 | M | Right kidney | Palpable mass | Simple excision | 7*8*10 | 210 | Oncocytoma | None found |
| **Current case** | 2009 | 25 | F | Left kidney | Abdominal mass | Radical Nephrectomy | 25*16*12 | 3380 | Oncocytoma | None found |

**Table S1:** The summary of the studies about giant oncocytoma published in the English literature
